# Visible and Near-Infrared Multispectral Features in Conjunction with Artificial Neural Network and Partial Least Squares for Predicting Biochemical and Micro-Structural Features of Beef Muscles

**DOI:** 10.3390/foods9091254

**Published:** 2020-09-08

**Authors:** Abderrahmane Aït-Kaddour, Donato Andueza, Annabelle Dubost, Jean-Michel Roger, Jean-François Hocquette, Anne Listrat

**Affiliations:** 1 VetAgro Sup, INRAE (National Institute for Agriculture, Food, and Environment), Université Clermont Auvergne, 63370 Lempdes, France; 2 Chem House Reasearch Group, 34060 Montpellier, France; jean-michel.roger@inrae.fr; 3 VetAgro Sup, UMR1213 Herbivores, INRAE, Clermont Université, Université de Lyon, 63122 Saint-Genès-Champanelle, France; donato.andueza@inrae.fr (D.A.); annabelle.dubost@inrae.fr (A.D.); jean-francois.hocquette@inrae.fr (J.-F.H.); anne.listrat@inrae.fr (A.L.); 4 UMR ITAP (Information-Technologies-Environmental Analysis-Agricultural Processes), INRAE (National Institute for Agriculture, Food, and Environment), Montpellier SupAgro, University Montpellier, 34060 Montpellier, France

**Keywords:** intramuscular lipids, meat, *perimysium*, endomysium, intramuscular connective tissue

## Abstract

The objective of this study was to determine the potential of multispectral imaging (MSI) data recorded in the visible and near infrared electromagnetic regions to predict the structural features of intramuscular connective tissue, the proportion of intramuscular fat (IMF), and some characteristic parameters of muscle fibers involved in beef sensory quality. In order to do this, samples from three muscles (*Longissimus thoracis, Semimembranosus* and *Biceps femoris*) of animals belonging to three breeds (Aberdeen Angus, Limousine, and Blonde d’Aquitaine) were used (120 samples). After the acquisition of images by MSI and segmentation of their morphological parameters, a back propagation artificial neural network (ANN) model coupled with partial least squares was applied to predict the muscular parameters cited above. The results presented a high accuracy and are promising (*R*^2^ test > 0.90) for practical applications. For example, considering the prediction of IMF, the regression model giving the best ANN model exhibited R^2^P = 0.99 and RMSEP = 0.103 g × 100 g^−1^ DM.

## 1. Introduction

Due to the different food crises (mad cow disease, adulterated milk in China, “horse gate” etc.) and consumer mistrust in the agro-food industry, the sector needs effective techniques to authenticate and determine food quality during processing. Different techniques have been proposed and used in practice by the industry for this purpose. Among these techniques, classical spectroscopic techniques (e.g., infrared) can be used to perform in-, on-, or at-line measurements, because they have the advantage of not being in physical contact with food products. This decreases the risks of sample physical alteration or contamination during the measurement, particularly for sensitive samples like meat. These techniques are also known to be generally quick, cost-effective, and environmentally safe. Therefore, they can be a remarkably effective alternative to traditional analytical techniques, which are generally expensive, technically long to implement, and require a highly skilled operator. Among these techniques, multispectral imaging (MSI) is able to depict both spectral and spatial features of a region of interest of samples with sizes ranging from a few square millimeters up to 10 cm^2^. This provides the possibility to collect better representative information on the sample because the distribution of biochemical components is generally heterogeneous and is not similar from one point to another. Compared with hyperspectral imaging, the lower processing time generally makes it easier for MSI to meet the speed requirements of industrial production lines [1]. The other advantage of MSI is its ability to record data at different positions in the electromagnetic spectrum [2,3], enabling the collection of very rich information about sample chemical characteristics (i.e., absorption, reflection, fluorescence) in spite of the small number of wavelengths. This technology has been used to authenticate and predict the physicochemical parameters of food products, such as cheeses [3,4] and pomegranate fruit [5].

Beef consumers are demanding a constant and high sensory quality of beef. Frequent and accurate determination of the quality of the beef (tenderness, flavor, juiciness) is therefore essential to guarantee to the consumer a meat of constant quality. The sensory quality of beef depends in great part on muscle characteristics [6]. The muscle (and, therefore, meat) is mainly composed of muscle fibers surrounded by connective tissue (CT). The latter is a three-dimensional network that is formed of two structures (*endomysium* and *perimysium*) commonly known as intramuscular CT (IMCT). *Endomysium* surrounds each muscle fiber individually, while *perimysium*, in which intramuscular fat (IMF) is located, surrounds groups of muscle fibers. The major components of IMCT are a set of fibrillar collagens (commonly called total collagen), the fibers and fibrils of which are linked together by covalent links called cross-links (CLs). This network of fibers and fibrils of collagen is wrapped in proteoglycans (PGs) [7].

IMCT’s structural and biochemical characteristics, as well as those of the muscle fibers and IMF, are known to be involved in beef sensory quality [8,9]. While MSI has been used to predict total collagen and IMF contents [4,10], this technique has never been used to predict the other muscle characteristics cited above in relationship with beef quality traits. Unlike hyperspectral imaging, MSI has been scarcely used on beef. However, it has previously been studied to predict the tenderness and mechanical properties of beef [10,11], and also to discriminate samples according to animal age and muscle type [2,4].

An artificial neural network (ANN) is an artificial intelligence tool. The ANN model is designed to predict the dependent variable (*Y*) automatically from the independent data set (*X*) presented during the training of the network processes [12]. A variety of ANN models are available, and the most commonly used one is the feed forward multi-layer perceptron (MLP) model. The feed-forward MLP consists of an input layer, an output layer and one or more hidden layers between them. Each neuron is completely connected to all the neurons in the next layer, but only forward links are available [13]. The MLP uses the back propagation learning algorithm, which aims to specify the best parameters to model the relationship between the input and output variables [14]. ANNs, among other data mining techniques, combined with spectral data, have been increasingly used in animal science (i.e., for the detection of mastitis, determination of culling reasons, prediction of milk yield during lactation) [15,16]. Nonetheless, the application of these methods to predict collagen and IMCT properties has, as far as we know, not been addressed in the literature. Therefore, the objective of this study was to determine the possibility of using MSI in the visible (VIS) and near-infrared (NIR) electromagnetic regions coupled with a back propagation ANN to predict both the composition (IMF, total and insoluble collagen contents) and some geometrical features of the structure of the IMCT (e.g., area, length and width of both the *perimysium* and the *endomysium*) and density of muscles fibers.

## 2. Materials and Methods

The study was carried out in compliance with French recommendations and those of the Animal Care and Use Committee of the National Institute for Agriculture, Food, and Environment (INRAE) of Clermont-Ferrand/Theix, France, for the use of experimental animals including animal welfare.

### 2.1. Beef Muscles

The experiment was performed with 40 young entire males slaughtered at 17 months of age (average live weight 670 kg) from three breeds of cattle (Aberdeen Angus (AA) (*n* = 12), Limousine (LI) (*n* = 14), and Blonde d’Aquitaine (BA) (*n* = 14). Three muscles (*Longissimus thoracis* (LT), *Semimembranosus* (SM), and *Biceps femoris* (BF)) were sampled from each animal in order to create variability in muscle biochemical traits. The conditions of the production of animal samples and the preparation of these samples for structural and biochemical analysis and MSI were previously described [2,8]. At the end of the sampling experiment, a total of 120 samples of beef muscles were obtained.

### 2.2. Structural and Biochemical Features of Connective Tissue

All the methods used herein were previously described in detail [16,17]. For structural characterization, stained cross-sections were analyzed by image analysis via two programs developed using Visilog 6.7 Professional Software (Noesis, Gif-Sur-Yvette, France). The binary images obtained with the programs were used to determine the area of the *perimysium* and *endomysium* (both expressed as percentage of the total image area). The skeletonized images obtained via the programs were used to determine the total length of the *perimysium* (expressed in mm × mm^−2^ of the image). For the muscle fiber study, the labelled images obtained with the programs were used to determine the fiber number per square millimeter. For biochemical characterization, total and insoluble collagen contents were determined according to the method of Woessner [18] updated by Listrat et al. [19]. For both total and insoluble collagen, data are expressed in milligrams of hydroxy-proline (OH-Prol) per gram of dry matter (mg OH-Prol × g^−1^ DM). The intra muscular fat (IMF) content (expressed in g × 100 g^−1^ DM) was determined on the one hand by the reference method of Folch et al. [20] based on biochemical extraction and filtration of fat [20] and on the other hand by NIR spectroscopy. For NIR spectroscopy, the statistical parameters (coefficient of determination and standard error of cross-validation) of the prediction model were 0.81 and 1.75 g × 100 g^−1^ DM, respectively.

### 2.3. Multispectral Imaging of Beef Muscles

MSI data were recorded from meat samples using a VideometerLab2^®^ (Videometer A/S, Herlev, Denmark). The device was equipped with a multispectral camera (Scorpion SCOR-20SOM, 1200 × 1200 pixels, Point Gray Research, Inc., Richmond, British Columbia, Canada) that enabled the recording of 19 images per sample in the VIS NIR region from 405 to 1050 nm [3,21]. Calibration of the camera was performed using three plates: a white one for reflectance correction, a dark one for background correction, and a dotted one for pixel position calibration. For each muscle and before image acquisition, the samples were defrosted at 4 °C in their plastic bag, equilibrated at 20 °C in a water bath, and then cut gently to 6 × 5 × 1.5 cm^3^ and wiped with paper towels in order to reduce the effect of reflection due to surface moisture. The samples were then placed in the dark by lowering the hollow sphere of the MSI system onto the sample support plate. Each muscle was illuminated successively by strobing the LEDs. Finally, a data cube for each muscle was obtained with 700 pixels in the *X* axis, 575 pixels in the *Y* axis (together called the image in the rest of this document), and 19 wavelengths in the *Z* axis. Two data cubes were recorded per muscle by performing an acquisition on both sides of the muscle, representing after the analysis 240 data cubes or 4560 images (i.e., 240 × 19).

### 2.4. Image Segmentation and Morphological Object Features

**Figure 1:**
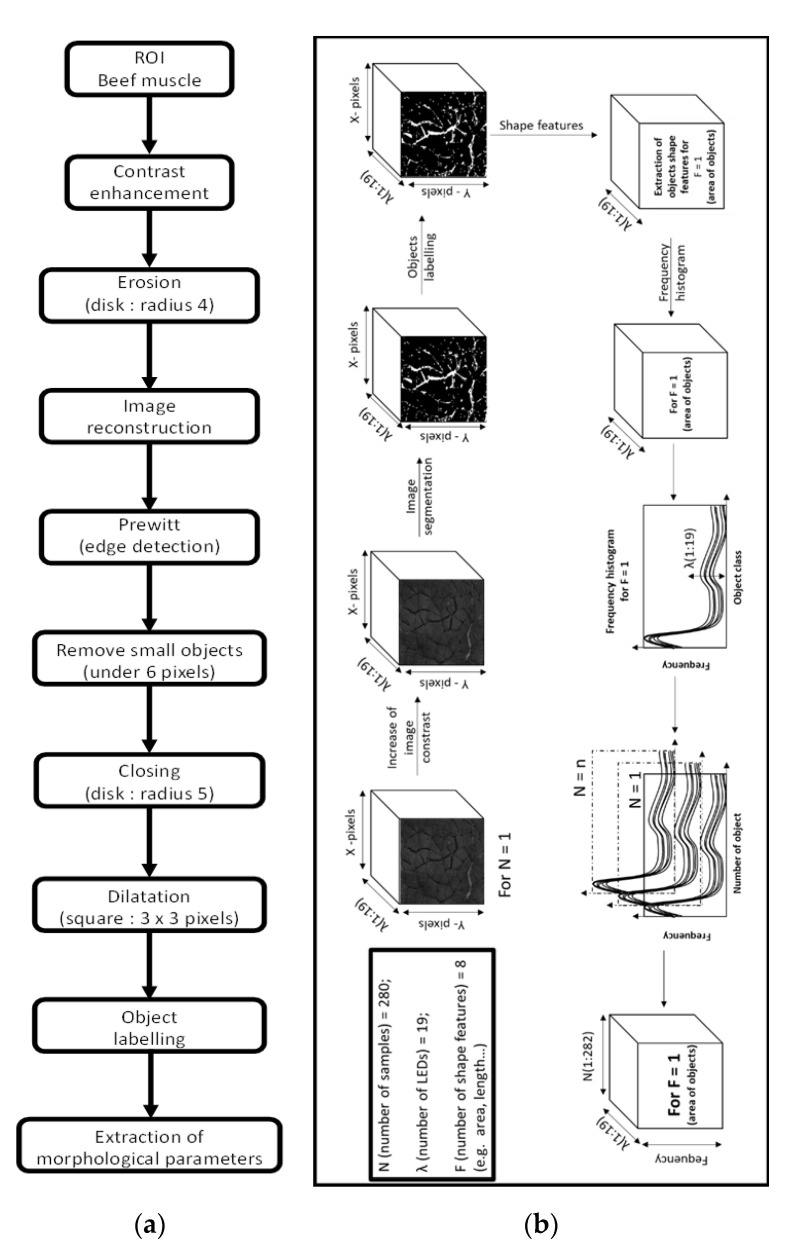
(**a**) Detailed schematic sequence for extraction of the shape parameters of meat marbling for one multispectral image and (**b**) sequence describing the organization of the histograms of the frequencies of the objects extracted from the dataset. ROI: region of interest; LED: Light-emitting diode.

**Figure 2:**
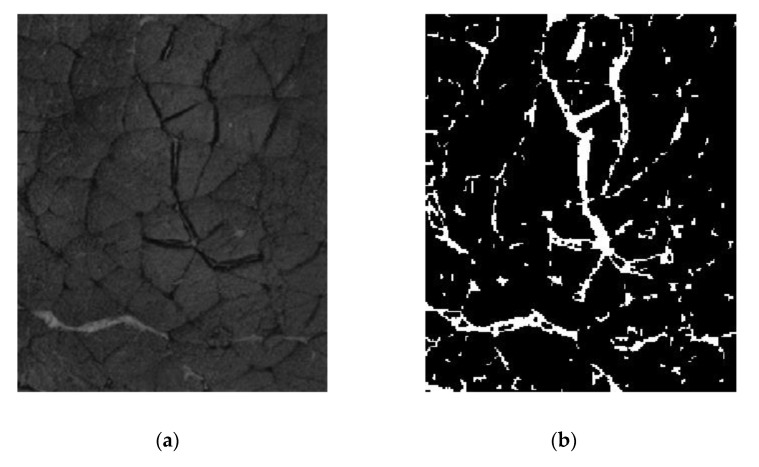
*A Biceps femoris* multispectral image recorded at 450 nm (**a**) before and (**b**) after the segmentation procedure.

The image correction and segmentation were performed by using the image processing toolbox for Matlab software R2012b (The MathWorks, Natick, MA, USA). Before image segmentation, the contrast of each image (i.e., recorded per wavelength) was highlighted individually by using the contrast-limited histogram equalization method [22]. The image was then processed in order to perform the segmentation that is needed for the extraction of the objects considered as being IMCT. A specific algorithm sequence was developed based on Prewitt edge detection and mathematical morphology, to target objects, which belong to the IMCT. Different processing operations were used in the algorithm in order to obtain the best IMCT parameters extraction, like erosion, dilatation and thresholding (Figure 1a). After edge detection, the geometrical characteristics of the image objects considered for IMCT were extracted from the binary images. Eight parameters were calculated: area (1), major axis length (2), minor axis length (3), eccentricity (4), orientation (5), solidity (6), and extent (7) and perimeter (8). Then, a classification of the objects by size into 10 classes (100, 200, 300, 400, 500, 600, 700, 800, 900 and 1000) between the minimum and maximum of the different object parameters was carried out to produce the characteristic histogram frequency parameter of each sample image. After extraction of the histogram frequency of the objects from each image, 10 new data cubes were obtained, one per histogram frequency class. Each cube presented in one dimension the number of samples (i.e., beef muscles), in the second one the size of the histogram frequency considered (i.e., class 100, 200, 300, 400, 500, 600, 700, 800, 900 or 1000), and in the last one the wavelength dimension (i.e., 19 LEDs). The detailed procedure is presented in Figure 1b. Furthermore, to demonstrate the performance of the developed segmentation procedure, an example of an image obtained after segmentation is presented in Figure 2.

### 2.5. Artificial Neural Network Design and Architecture

The ANN design has been developed using the neural network toolbox in Matlab. A multilayer feed-forward network was selected while the back-propagation-training algorithm was applied to train the network. Uploading the entire image to the network would require thousands of components of the vector and, consequently, the same number of input neurons in the generated ANN. The learning process of a network with a large number of input neurons (and similar numbers of neurons in the hidden layers) would require a large number of learning examples, which, in turn, would increase the requirements in terms of computer processing capacity [23,24,25,26]. For this reason, in the present study, instead of directly using the histograms of the extracted morphological objects as input variables in the ANN model, we transformed them into synthetic variables by partial least squares (PLS).

PLS has the advantage of taking into account the correlation between the *X* and *Y* matrices, while extracting the latent variables (LVs) from the *X* matrix. In this way, the LVs directly refer to the given component [27], which is unlikely in principal components analysis (PCA). Before performing PLS, the histograms were normalized (area under the curve made equal to 1) and mean-centered. Ten LVs, for each *Y* parameter to predict (i.e., biochemical and geometrical features of the structure of the IMCT of beef muscles), were chosen because they gathered more than 90% of *X* data variability. A feed-forward neural network trained by back-propagation was calculated using the nftool in Matlab. The neurons were organized into four layers: one input layer, two hidden layers, and one output layer. There were as many input neurons as LVs (*n* = 10) and one output neuron. Concerning the two hidden layers, different networks were tested by varying the number of neurons from 2 to 8 in each hidden layer and testing each possible combination (8 × 8 = 64). Moreover, different numbers of training epochs (from 0 to 1000) were tested. In the training sequence, the output of the network was compared to known values, and errors were back-propagated to the hidden and input layers to adjust the weights and minimize the root mean squared error step by step using the method of Levenberg Marquardt. The procedure was repeated until the errors between the output and known values were minimized. Before performing PLS-ANN, the initial data set was subdivided into three data tables: a training dataset containing 70% of the observations (*n* = 168), a validation data set containing 15% of the observations (*n* = 36), and a testing dataset containing the remaining data (*n* = 36). The data division was performed randomly by using the function divider provided in the nftool for Matlab.

In order to assess the performance of the models calculated, the determination coefficients for calibration (R^2^C), validation (R^2^V), and prediction (R^2^P) and their corresponding root mean square errors for calibration (RMSEC), validation (RMSEV), and prediction (RMSEP) were calculated. In detail, *R*^2^ indicates the proportion of the variance of the predicted variable (*Y*) that can be explained by the variance of the independent variable (*X*), and the values of RMSEC, RMSEV, and RMSEP evaluate the fitting degree of regression during calibration, validation, and prediction. Generally speaking, a robust model should have high values of R^2^C, R^2^V, and R^2^P close to 1, and low values of RMSEC, RMSEV, and RMSEP close to 0.

## 3. Results and Discussion

### 3.1. Data Sets

Table 1 presents the biochemical and microstructural characteristics of the muscles. These results were previously presented and discussed in detail [17]. Briefly, variability was larger for total lipid content and collagen contents. More specifically, the total collagen content of the studied muscles varied from 2.94 to 10.45 mg OH-prol × g^−1^ DM, while the insoluble collagen content varied from 2.01 to 6.87 mg OH-prol × g^−1^ DM. The max and min values obtained for IMF content varied from 3.61 to 22.82 g × 100 g^−1^ DM respectively. Among the muscles’ microstructural characteristics, the variability of *perimysium* features was larger than that of the other microstructural parameters. These results are coherent with a previous investigation [6] reporting that the perimysial collagen of muscles ranged from 0.5% to 4.8%, whereas endomysial collagen varied from 0.5% to 1.2%. The samples presented a *perimysium* with an average area varying from 3.8 to 16.1% and a length varying from 9.38 to 34.61 mm × mm^−2^ of the image. For the *endomysium*, average area values of 3.1 to 10.2% were observed with average length varying from 2.42 × 10^−2^ to 3.55 mm. For the fiber density, we observed min and max values of 202.9 to 441.8 fibers per mm^2^.

### 3.2. Multispectral Image Absorbance Peaks

**Figure 3:**
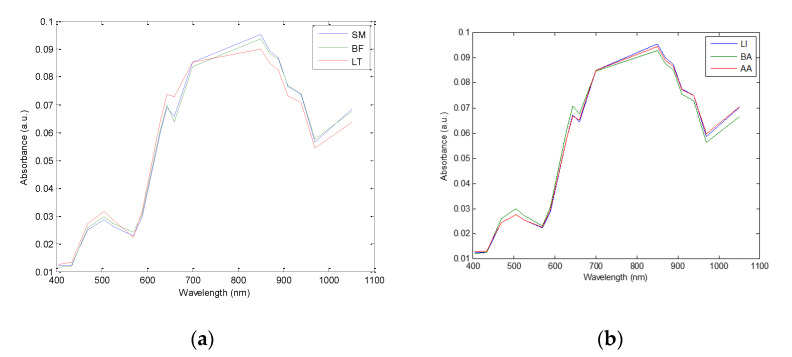
(**a**) Mean spectra of the three muscles; *Biceps femoris* (BF), *Longissimus thoracis* (LT), and *Semimembranosus* (SM) of the Blonde d’Aquitaine breed and (**b**) mean spectra of the three breeds; Aberdeen Angus (AA), Blonde d’Aquitaine (BA), and Limousine (LI) considering all three muscles.

Mean normalized spectra (with an area under the curve equal to 1) of the three different muscles (BF, LT, and SM) for BA breed individuals and the mean of the three different breeds (AA, BA, and LI) considering all the three muscles are presented in Figure 3a,b, respectively. All the spectra present two broad peaks: a small one with a maximum at 500 nm and a higher one exhibiting a shoulder at 645 nm and a maximum at 850 nm. The three muscles investigated (Figure 3a) presented differences from 405 and 700 nm (i.e., in the VIS region) certainly due to differences in the muscles’ color [28,29,30,31,32,33,34,35,36]. Torrescano et al. [35] and Isdell et al. [36] reported that SM and BF exhibit similar colors, different to that of LT muscle. Spectral differences observed in the NIR region, between 850 and 1050 nm, can be related to differences in biochemical and structural differences from one muscle to another [17,37]. Bands at 850, 870, and 890 nm can be assigned to O-H vibration of water molecules. The spectral bands located between 910 and 970 nm are related to C-H and O-H vibrations of both proteins and water [38,39,40,41]. Finally, the band at 940 nm can be related to meat fat and the 1050 nm band to both fat and proteins [42,43,44]. These observations corroborate those of Bureš et al. [37], who noted significant differences between LT (0.46–0.498 g/100 g) and SM (0.368–0.389 g/100 g) in terms of total fatty acids. Moreover, Dubost et al. [17] reported a higher total lipid content in LT muscles (8.46 g × 100 g^−1^ DM) compared to SM and BF muscles (6.70 and 7.65 g × 100 g^−1^, respectively). Furthermore, they reported that SM and BF muscles are close in their structural (e.g., *perymisium* area, *endomysium* area, branch points) characteristics while the LT muscle is different.

Concerning the breeds, in the VIS region (i.e., from 405 to 700 nm), LI and BA presented equivalent absorbance, while AA exhibited slightly higher absorbance values. In the NIR region (i.e., from the 700 to 1050 nm), the opposite was observed (Figure 3b). These results certainly depict differences in muscle metabolism between the three breeds. Indeed, the muscles of animals such as AA are more oxidative and less glycolytic than those of animals such as those reported by Bonnet et al. [45]. The muscles of BA are more glycolytic and consequently less red than AA muscles, which are more oxidative. It has also been reported that late-maturing cattle breeds (such as BA) deposit more muscle and less fat, compared to early-maturing cattle (such as AA) [17,46].

### 3.3. Artificial Neural Network Results

**Figure 4:**
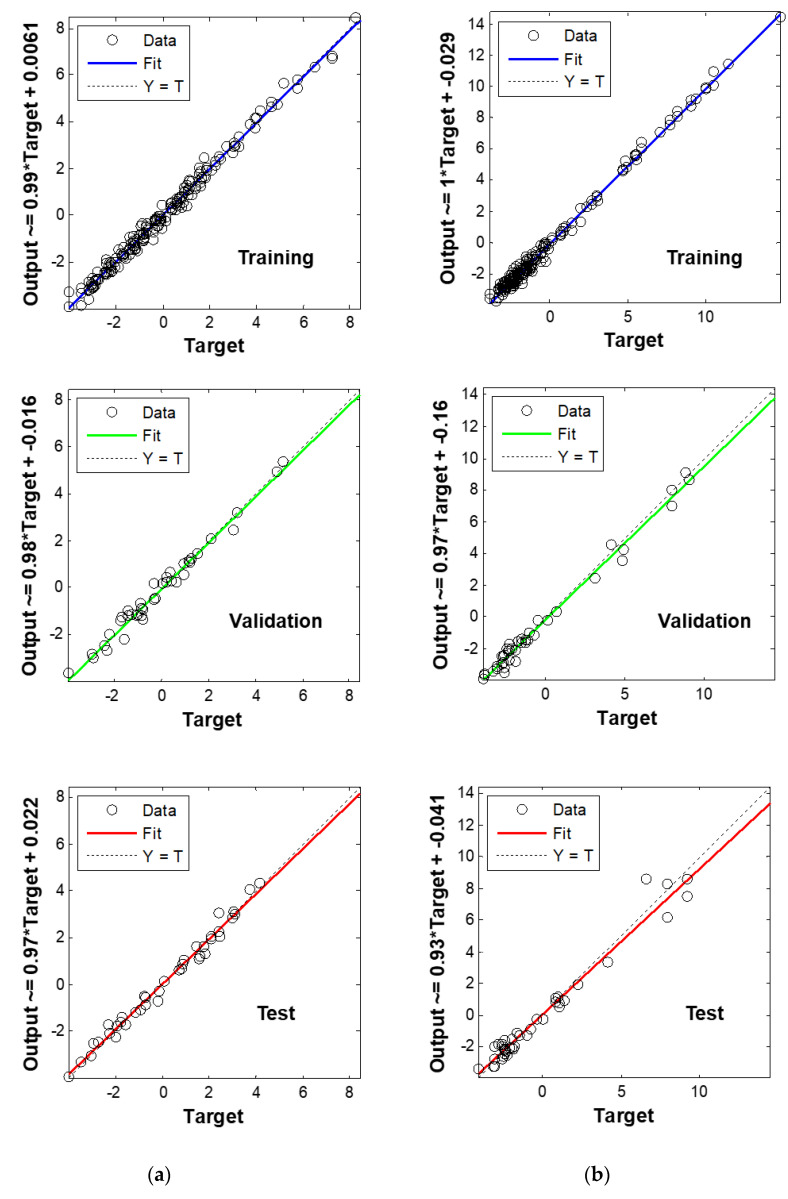
Partial least squares (PLS)-artificial neural network (ANN) regression curves of target value (X) versus output values (Y) of the (**a**) *Perimysium* area (%) and (**b**) intramuscular fat (g × 100 g^−1^ DM) parameters obtained for training, validation, and test steps of the models.

The ANN statistics corresponding to the best models are summarized in Table 1 and the Figure 4 illustrates the linear regression models’ predicted and measured values for the *perimysium* area and IMF parameters. The critical step for an accurate regression model is to select the optimum factors (numbers of epoch, neurons, etc.) needed to design the best model. In the present study, in order to select the optimum ANN factors, we firstly identified the optimum numbers giving the minimum RMSEP for each histogram frequency class (i.e., 100 to 1000). Secondly, the different models based on each histogram frequency were compared and the best one was chosen based on the lowest RMSEP factor.

A global overview of the results (Table 1) highlighted that all the ANN models were accurate because the R^2^P values were higher than 0.96. Moreover, the MSI features enabling us to reach these results are the orientations of objects when considering the 900 or 800 histogram frequency. Considering the prediction for IMF, the regression model giving the best ANN model exhibited an R^2^P value of 0.99 and RMSEP of 0.10 g × 100 g^−1^ DM (Table 1). Our results were more accurate when compared to those of Ballerini et al. [47], who also used a segmentation algorithm of muscle from fat developed on the basis of their characteristics in three-dimensional color space. A correlation value of 0.79 was obtained between the chemical analysis and fat percentage computed by image segmentation. The calibration model constructed by Abouelkaram et al. [48] (R^2^CV = 0.86) also presented lower accuracy. This difference can be related to the small number of excitation light sources used to record muscle images and to the low variability in muscle databases previously used. Indeed, during their studies, the previous authors used only three excitation light sources (white Xenon lamp and UV lights at 320 and 380 nm) on BF muscles coming from six animals. Nonetheless, in another study, El Jabri et al. [10], using equivalent excitation light sources on a larger sample database, also reported lower accuracy after cross-validation (R^2^CV = 0.76). The database included only SM muscles. Moreover, the muscles were taken from two breeds, Salers (*n* = 12) and Holstein (*n* = 12) cull cows. Du et al. [49] reported, after cross-validation, an R^2^ value of 0.69, when considering 50 steaks taken from one muscle (*Longissimus dorsi*) from four breeds, namely, Charolais cross-breed, pure Charolais, AA, and Belgian Blue. The steak images were captured using four fluorescent lamps with plastic light diffusers and a polarizer, and they were saved in three-dimensional RGB (red, green, and blue) color space. Those results suggest that the superior performance of our predictive models can certainly be explained by the original method (i.e., ANN) we used. Indeed the aforementioned studies used multi linear regression or partial least squares regression, which are more adapted to giving models a good accuracy when a linear relationship exists between the *X* and *Y* data. It is established that ANNs are generally more accurate for modeling nonlinear relationships between *X* and *Y*. Nonetheless, the segmentation procedure performed on the MSI data in the present study also has the potential to increase the accuracy of the models. Considering the total collagen content, the best model presented an R^2^P value of 0.99 with an RMSEP of 0.02 mg OH-pro × g^−1^ DM. This model was calculated with six neurons in both the first and second layers of the ANN. Compared to our results, El Jabri et al. [10] and Abouelkaram et al. [48] reported lower accuracy for total collagen: *R*^2^ = 0.74 and 0.86, respectively. For the insoluble collagen content, the best predictive model gave an R^2^P value of 0.99 and an RMSEP of 0.007 mg OH-pro × g^−1^ DM. Compared to that for total collagen, the ANN model for insoluble collagen was obtained with a lower number of neurons in the second layer (4 vs. 6) and a lower number of epochs (6 vs. 44). The best predictive accuracy reported in the present study was for total collagen. When compared to the literature, this can be explained by the same factors aforementioned for the IMF: (i) high database variability by considering three types of animals (LI, BA, and AA) and three muscle types (LT, SM, and BF); (ii) differences in excitation lights (i.e., visible and NIR vs. Xenon lamp and UV); and (iii) differences in segmentation algorithms used for spectral image processing. Considering the *perimysium* and endomysium geometrical features (area, length, and width), the best predictive models gave an R^2^P value higher than 0.96, indicating good accuracy (Table 1). Concerning the prediction of the muscle fibers density, the ANN models presented an R^2^P value of 0.98.

This study brings new insights concerning the prediction of both total and insoluble collagen contents as well as the microstructural characteristics of the *endomysium* and *perymisum* (e.g., area, length) and fiber density of beef muscle, by using the MSI technique. The reported results are promising due to the high R^2^P values and because those muscle characteristics can be related to meat tenderness despite contradictory results such as those by Chriki et al. [50], who showed that muscle biochemical traits explain only a small variability of beef tenderness. However, Chriki et al. [50] did not include any characteristics of *endomysium* and *perimysium* such as their thickness in their prediction model. Indeed, a potential role of thickness of the muscle *perimysium* in tenderness has been reported by various authors [51,52,53]. For example in raw pork [50,51,52,53], a positive relationship between perimysial thickness and shear force (*R*^2^ = 0.96 and 0.56, respectively) was found, while Dubost, et al. [8] reported a negative contribution of *perimysium* and *endomysium* surface areas to tenderness.

## 4. Conclusions

This study highlighted that a spectral signature in meat samples does exist. We showed that this signature can be related to the microstructural characteristics of meat since multispectral imaging can gather the subtle physical and chemical features of bovine muscles. We also demonstrated that the prediction of IMF content, IMCT structural features, and some muscle fiber characteristics is feasible with good accuracy using the proposed technology. The reported results are very promising, given that predicting muscle characteristics using multispectral imaging may be a good way to replace conventional biochemical or physical methods.

This work also highlighted for the first time the potential of a VIS-NIR MSI technology combined with ANN analysis to predict both the chemical and microstructural characteristics of meat. This was performed in order to propose, in the long run, a non-destructive, fast, and low-cost technique to be implemented by the meat industry with the ultimate objective to better monitor and predict beef eating quality.

## Figures and Tables

**Table 1:** The best artificial neural network (ANN) results obtained for structural parameters of intramuscular connective tissue and biochemical features of the beef muscles.

Microstructural and Biochemical Parameters	Min	Max	Mean	SD ^1^	CV ^1^ (%)	Number of Classes in the Histogram	MSI ^1^ Features	N1L ^1^	N2L ^1^	Epoch	R2C ^1^	R2V ^1^	R2P ^1^	RMSEC ^1^	RMSEV ^1^	RMSEP ^1^
*Perimysium* area (% of the total image area)	3.8	16.1	8.0	1.9	23.3	900	Orientation	2	6	10	0.99	0.98	0.99	0.050	0.076	0.058
*Perimysium* length (mm ^×^ mm^−2^ of the image)	9.38	34.61	18.66	3.86	20.6	900	Orientation	2	6	37	0.99	0.99	0.98	0.325	0.298	0.286
*Perimysium* width (µm)	0.00	5.2 × 10^−3^	4.24 × 10^−3^	2.72 × 10^−4^	6.4	900	Orientation	6	8	12	0.97	0.96	0.96	0.000	4.1 × 10^−9^	3.04 × 10^−9^
*Endomysium* area (% of the total image area)	3.1	10.2	5.8	1.0	17.2	900	Orientation	8	8	53	0.99	0.99	0.98	0.015	0.018	0.018
*Endomysium* length (mm × mm^−2^ of the image)	2.42 × 10^−2^	3.55 × 10^−2^	3.00 × 10^−2^	2.09 × 10^−3^	7.0	900	Orientation	8	2	11	0.98	0.98	0.98	1.01 × 10^−7^	8.18 × 10^−8^	8.11 × 10^−8^
*Endomisium* width (µm)	1.14	2.8	1.94	0.30	15.4	900	Orientation	8	6	13	0.99	0.99	0.99	0.001	0.001	0.001
Fibers density (number mm^−2^)	202.9	441.8	308.4	43.3	14.0	800	Orientation	2	4	20	0.98	0.97	0.98	46.48	55.39	34.28
Total collagen (mg OH-Pro × g^−1^ DM *)	2.94	10.45	5.59	1.32	23.6	900	Orientation	6	6	44	0.99	0.99	0.99	0.021	0.024	0.023
Insoluble collagen (mg OH-Pro × g^−1^ DM *)	2.01	6.87	3.74	0.83	22.2	900	Orientation	6	4	6	0.99	0.98	0.99	0.009	0.017	0.007
IMF * (g × 100 g^−1^ DM)	3.61	22.82	7.60	3.00	39.4	900	Orientation	6	4	13	0.99	0.99	0.99	0.111	0.140	0.103

^1^ OH-prol: hydroxy-proline; DM: dry matter; IMF: intra muscular fat; SD: Standard deviation; CV: coefficient of variation (%); MSI: multispectral Image; N1L and N2L: number of neurons in the first and second layer; R^2^C, R^2^V, R^2^P: coefficient of correlation for calibration, validation, and prediction, respectively; RMSEC: root mean square error of calibration; RMSEV: root mean square error of validation; RMSEP: root mean square error of prediction. * DM: dry matter.
